# Persistent arthralgia and related risks factors in laboratory-confirmed cases of Chikungunya virus infection in Mexico

**DOI:** 10.26633/RPSP.2017.72

**Published:** 2017-04-14

**Authors:** Efrén Murillo-Zamora, Oliver Mendoza-Cano, Benjamín Trujillo-Hernández, Ramón Alberto Sánchez-Piña, José Guzmán-Esquivel

**Affiliations:** 1 University of Colima, School of Medicine Doctorate in Health Science program Colima Mexico University of Colima, School of Medicine, Doctorate in Health Science program, Colima, Colima, Mexico.; 2 University of Colima, School of Civil Engineering, Coquimatlan University of Colima, School of Civil Engineering, Coquimatlan Colima Mexico University of Colima, School of Civil Engineering, Coquimatlan, Colima, Mexico.; 3 University of Colima, School of Medicine, Colima University of Colima, School of Medicine, Colima Colima Mexico University of Colima, School of Medicine, Colima, Colima, Mexico.; 4 Harvard University, T.H. Chan School of Public Health Center for Health and the Global Environment BostonMassachusetts United States of America Harvard University, T.H. Chan School of Public Health, Center for Health and the Global Environment, Boston, Massachusetts, United States of America.; 5 Mexican Institute of Social Security Clinical Epidemiology Research Unit ColimaMassachusetts Mexico Mexican Institute of Social Security, Clinical Epidemiology Research Unit, Colima, Colima, Mexico.

**Keywords:** Chikungunya fever, cohort studies, risk groups, arthralgia, chronic pain, Mexico, Fiebre chikungunya, estudios de cohortes, grupos vulnerables, artralgia, dolor crónico, México, Febre de chikungunya, estudos de coortes, grupos de risco, artralgia, dor crónica, México

## Abstract

**Objective.:**

*To estimate the cumulative incidence of persistent arthralgia at 6 months from acute Chikungunya virus (CHIKV) infection and to evaluate the association of clinical markers with the risk of long-term arthralgia*.

**Methods.:**

*This multicenter retrospective cohort study was conducted in the Mexican state of Colima. A total of 136 individuals aged 15 years and older with serologically confirmed CHIKV infection were enrolled. Participants were interviewed at 6 months from the onset of symptoms, and self-reported persistent arthralgia (PA) was the main binary outcome. A self-report numeric rating scale (NRS) ranging from 0 to 10 was used to estimate the severity of articular pain*.

**Results.:**

*The cumulative incidence of PA was 41.9%. Severe pain (NRS ≥ 7) presented in 36.8% of participants with PA. In multiple analysis, individuals aged 40 years and older (risk ratio (RR) = 1.60; 95% confidence interval (CI), 1.03-2.48) and those with articular pain at 3 months post-infection (RR = 3.95; 95% CI, 1.95-8.01) had a significantly increased risk of PA at 6 months from CHIKV infection*.

**Conclusions.:**

*To the best of our knowledge, this is first report of a CHIKV-associated longterm outcome in Mexico, where the incidence of the infection has been high. This is also the first study in Latin America evaluating several factors associated with the risk of PA. Our findings may be useful in health care settings to stratify the risk of chronic arthralgia secondary to CHIKV infection and to identify patients who would benefit clinically from early medical intervention*.

## RESUMEN

**Objetivo.:**

Calcular la incidencia acumulada de artralgia persistente en los 6 meses siguientes a la infección aguda por el virus del Chikunguña y evaluar la asociación entre los marcadores clínicos y el riesgo de artralgia a largo plazo.

**Método.:**

Este estudio de cohortes multicéntrico y retrospectivo se condujo en el estado mexicano de Colima con la participación de un total de 136 personas de al menos 15 años de edad con infección aguda por el virus de Chikunguña serológicamente confirmada. Se entrevistó a los participantes en los seis meses posteriores al inicio de los síntomas, y la artralgia persistente autonotificada fue el principal resultado dicotómico. Para calcular la gravedad del dolor articular, se empleó una escala de clasificación numérica, en que el participante puntuaba la gravedad del dolor articular de 0 a 10.

**Resultados.:**

La incidencia acumulada de artralgia persistente fue de 41,9%. De los participantes con artralgia persistente, 36,8% presentaron un dolor intenso (igual o mayor a 7 en la escala de clasificación numérica). En varios análisis, las personas de al menos 40 años de edad (razón de riesgo [RR] = 1,60; intervalo de confianza [IC] de 95%, 1,03-2,48) y las personas que experimentaban dolor articular en los tres meses posteriores a la infección (RR = 3,95; IC de 95%, 1,95-8,01) tenían un riesgo significativamente mayor de padecer artralgia persistente en los seis meses siguientes a la infección aguda por el virus del Chikunguña.

**Conclusiones.:**

En la medida de nuestro conocimiento, este es el primer informe acerca de un resultado a largo plazo asociado a la infección aguda por el virus del Chikunguña en México, donde se ha registrado una incidencia alta de la infección. También es el primer estudio en el que se evalúan varios factores asociados al riesgo de artralgia persistente realizado en América Latina. Nuestros resultados pueden ser útiles en los establecimientos de atención de salud para estratificar el riesgo de padecer artralgia crónica subsecuente a la infección aguda por el virus de Chikunguña e identificar a los pacientes que se beneficiarían clínicamente de una intervención médica temprana.

## RESUMO

**Objetivo.:**

Calcular a incidência acumulada de artralgia persistente após 6 meses de infecção aguda pelo vírus Chikungunya (CHIKV, sigla em inglês) e avaliar a associação de marcadores clínicos com o risco de artralgia a longo prazo.

**Métodos.:**

Métodos. Este estudo de coorte retrospectivo e multicêntrico foi realizado no estado mexicano de Colima. Um total de 136 indivíduos com idade a partir de 15 anos e infecção pelo CHIKV confirmada por sorologia foram incluídos. Os participantes foram entrevistados 6 meses após o surgimento dos sintomas. O desfecho binário principal foi artralgia persistente (AP) autorrelatada. Uma escala numérica autorrelatada de 0 a 10 foi utilizada para calcular a gravidade da artralgia.

**Resultados.:**

Resultados. A incidência acumulada de AP foi 41,9%. Dor intensa (≥ 7 na escala numérica) foi relatada por 36,8% dos participantes com PA. A análise múltipla revelou risco significantemente elevado de AP 6 meses após infecção pelo CHIKV nos indivíduos com idade igual ou superior a 40 anos (razão de risco (RR) = 1,60; intervalo de confiança (IC) de 95% = 1,03–2,48) e naqueles com dor articular 3 meses após a infecção (RR = 3,95; IC 95% = 1,95–8,01).

**Conclusões.:**

Até onde sabemos, este é o primeiro relato de um desfecho a longo prazo associado á infeccao por CHIKV no México, país onde a incidencia desta infeccao foi elevada. Este é também o primeiro estudo realizado na América Latina a avaliar vários fatores associados com o risco de AP. Nossos achados talvez sejam úteis para estratificacao do risco de artralgia crónica secundária á infeccao pelo CHIKV e para identificacao de pacientes nos quais intervencao médica precoce poderia ser benéfica do ponto de vista clínico.

Chikungunya virus (CHIKV) is transmitted to humans by the bite of infected *Aedes (Ae.) aegypti* and *Ae. albopictus* mosquitoes (1). The CHIKV-associated burden of disease represents a major challenge for health systems in tropical and subtropical areas, and there has been a global rise in these infections (2-5).

The autochthonous transmission of CHIKV in Mexico was first reported in October 2014 (6), and in the first quarter of 2015, local outbreaks occurred in the state of Colima, which is in the western part of the country. Colima is a subtropical area, with a total population of 650 000 inhabitants, as well as a permanent presence of *Ae. aegypti* and the consequent transmission of related vector-borne viral diseases (7, 8). Over the course of 2015, nearly 2.5% of the total population of the state was reported as suspected cases of acute illness in the Web-based National Epidemiological Surveillance System *(Sistema Nacional de Vigilancia Epidemiológica,* SINAVE) (9). Created in 1995, SINAVE is coordinated by the federal General Directorate of Epidemiology. SINAVE’s main objective is the timely identification of potential risks to public health from communicable and noncommunicable diseases (10). Nevertheless, there may be some underreporting of CHIKV cases in Mexico, as was true with an outbreak in India (11).

Acute infection is symptomatic in approximately 95% of individuals, and it is characterized by abrupt fever (> 39 °C) and severe polyarthralgia or arthritis (12). In most of the cases, the disease is self-limited. With the exception of arthralgia, recovery usually occurs after 7 to 10 days from the onset of symptoms (13, 14). Persistent arthralgia (PA) is a long-term outcome of CHIKV infection, and it is characterized by episodic relapse and recovery periods of articular pain. This may occur in > 70% of cases, and it may be highly incapacitating (15, 16).

The current scientific data regarding factors associated with increased risk of PA among individuals with a history of laboratory-confirmed CHIKV infection is limited, and most of the published studies have been conducted on the island of Réunion, which is an overseas department of France located in the Indian Ocean (15–20). To the best of our knowledge, there is only one published study evaluating CHIKV chronic manifestations in a large sample of individuals in a Latin American country (21), and it analyzed a limited number of clinical markers.

The aim of our study was to estimate the cumulative incidence of PA among adult individuals at 6 months from laboratory-confirmed CHIKV infection. In addition, we evaluated the association of several clinical markers with the risk of long-term articular pain.

## MATERIAL AND METHODS

### Study design

This multicenter retrospective cohort study was conducted in the state of Colima, Mexico, from December 2015 to March 2016. Serologically confirmed (reverse transcription quantitative-polymerase chain reaction (RT-qPCR)) cases of CHIKV infection with acute disease onset between July and September 2015 were included. Eligible cases were reported on the Web-based SINAVE by primary care public health care facilities, including with a telephone number that the patient had given to the physician. The medical units in our study location belong to the Mexican Institute of Social Security *(Instituto Mexicano del Seguro Social,* IMSS). The IMSS has 11 primary care medical units strategically situated to cover all the urban and rural areas of the state of Colima. (The IMSS, with the Health Secretariat (SS, *Secretaría de Salud)* and the Institute of Security and Social Services of the State Workers (ISSSTE, *Instituto de Seguridad y Servicios Sociales de los Trabajadores del Estado)* make up the Mexican public health care system).

### Participants

Individuals aged 15 years and older were enrolled. Patients with a self-reported history of systemic rheumatologic disease (rheumatoid arthritis, multiple sclerosis, or systemic lupus erythematous) or those who could not be contacted by telephone (e.g., incorrect phone number) were excluded.

A simple random procedure was used to select 150 individuals from subjects meeting the eligibility criteria. Potentially eligible individuals were invited by telephone to visit the nearest participating health care setting to be interviewed. The data from 136 serologically confirmed cases were analyzed.

### Data collection

Demographic and clinical information was extracted by the researchers from the database of the SINAVE surveillance system (after the application of a questionnaire by a physician) and then analyzed.

Practicing family physicians who had received standardized training used an interview questionnaire to obtain additional data on a number of topics. These included: days elapsed from disease onset to seeking medical attention; ambulatory medical management (yes/no); acute disease signs and symptoms (duration of acute disease, days; specific sites of arthralgia and articular effusion; associated signs and symptoms); self-reported arthralgia (any site) at 3 months from acute illness (yes/no); personal pathologic history (self-reported type 2 diabetes mellitus, high blood pressure, osteoarthritis, hyperuricemia; yes/no); outcome of interest (self-reported arthralgia after 6 months from acute illness, yes/no); and specific sites of arthralgia after 6 months from acute illness, if applicable.

In addition, a self-report numeric rating scale (NRS), ranging from 0 to 10, was used to evaluate the articular pain intensity.

### Outcome

Self-reported PA at 6 months from acute disease onset was the main outcome (yes/no). Two previously validated questions were used (20). The first question was: “Do you feel that you have made a complete recovery from articular manifestations since being diagnosed with a CHIKV infection?” (yes/no). The individuals who responded negatively were asked a second question: “Over the past eight days, did symptoms of CHIKV illness subside and subsequently recur?” Patients classified as PA-positive were those who stated they did not have a complete recovery from articular manifestations since the diagnosis of the viral infection and reported the recurrence of illness symptoms (arthralgia) over the preceding eight days (20).

### Laboratory methods

RT-qPCR analysis (QuantiTect, QIAGEN, Hilden, Germany) was performed in serum samples from participants, according to the manufacturer’s specifications (22). The venous blood samples were obtained within ≤ 5 days from the onset of signs and symptoms. The serological testing was carried out at the Division of Laboratories for Surveillance and Epidemiological Research *(División de Laboratorios de Vigilancia e Investigación Epidemiológica)* of the IMSS.

### Ethical considerations

This study was approved by the National Commission on Scientific Research *(Comisión Nacional de Investigación Científica)* (R-2016-785-004). Written informed consent was obtained from all participants before they were interviewed. In order to guarantee the anonymity of research participants, all identifying variables (name, health-insurance number, and contact phone number) were omitted, and an alphanumeric code was assigned to each individual.

### Data analysis

Summary statistics were employed to describe the study population, and the corresponding arithmetic means (± standard deviations) and proportions were calculated. The association of clinical markers with the binary outcome was evaluated with relative risk (RR) by means of generalized linear models. The statistical analysis was performed using the Stata/MP 13.0 statistical package (StataCorp, College Station, Texas, United States), and the significance level was set at 5%.

## RESULTS

**FIGURE 1:**
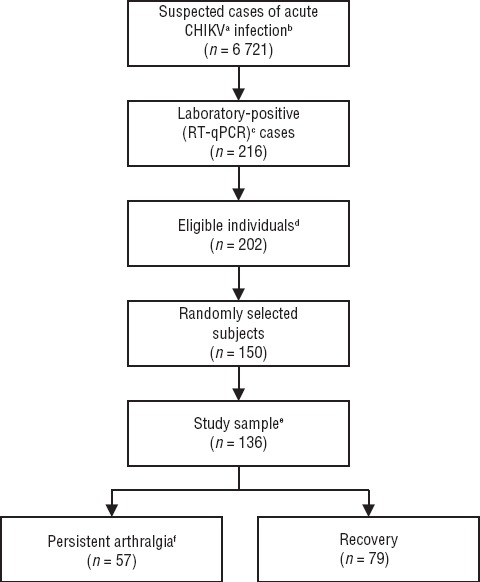
Profile of study of persistent arthralgia and related risks factors in laboratory-confirmed cases of Chikungunya virus infection, Mexico, 2015

***Source***: This figure was prepared by authors from the results of research.

^a^ CHIKV= Chikungunya virus.

^b^ Cases (abrupt fever > 39 °C and severe polyarthralgia or arthritis) reported by a participating medical unit, in the Web-based National Epidemiological Surveillance System from July to September 2015.

^c^ RT-qPCR = reverse transcription quantitative-polymerase chain reaction.

^d^ From the 216 laboratory-positive cases, the following were then excluded: age < 15 years old (*n* = 11), no contact phone number (*n* = 3).

^e^ From the 150 randomly selected subjects, the following subjects were then excluded: self-reported history of systemic lupus erythematous (*n* = 1), unable to be contacted by phone call (*n* = 8), unwilling to participate (*n* = 5).

^f^ Persistent arthralgia = episodic relapse and recovery periods of arthralgia and recurrence of articular pain over the past eight days before being interviewed.

The study profile is shown in Figure 1. Data from 136 laboratory-positive cases of CHIKV infection were analyzed. The mean interval between disease onset and the date of the interview was 181.3 ± 4.4 days. The characteristics of the study sample for selected variables are presented in Table 1. A large majority of the participants (89.0%) were between 15 and 64 years old. The overall cumulative incidence of PA at 6 months from acute Chikungunya infection was 41.9% *(n* = 57); the sex-stratified incidence was 31.8% in males and 46.7% in females *(P* = 0.099).

When compared with the participants who reported full recovery from articular pain, the individuals with self-reported PA had a significantly higher prevalence in relation to age ≥ 40 years (64.9% vs. 45.6%) and persistent articular pain at 3 months from acute disease onset (87.7% vs. 44.3%) (Table 2). The most frequent sites of rheumatologic manifestations among individuals with PA were the hands (59.6%), feet (59.6%), and ankles (57.9%). Severe pain (NRS ≥ 7) was self-reported by 36.8% (*n* = 21) of the participants with PA. No statically significant difference between individuals who self-reported PA and those with full recovery was found in terms of sex, mean age, severity of articular pain at illness onset and its duration (days), number of painful joints, presence of joint effusion, associated symptomatology (rash, headache, gastrointestinal manifestations, and fatigue), self-reported chronic noncommunicable diseases (type 2 diabetes mellitus, high blood pressure, and osteoarthritis), or hyperuricemia.

In multiple analyses (Table 3), clinical markers associated with increased risk of PA in laboratory-confirmed cases of CHIKV infection were age ≥ 40 years (RR = 1.60; 95% CI, 1.03-2.48) and self-reported persistent articular pain at any site after 3 months from acute disease onset (RR = 3.95; 95% CI, 1.95-8.01).

## DISCUSSION

After the first illness outbreak in the state of Colima, we found that nearly 42% of adult individuals with a history of laboratory-confirmed CHIKV infection reported chronic manifestations that consisted of episodic relapse-and-recovery periods of arthralgia. Our findings also suggest an increased risk of PA among individuals aged 40 years and older at disease onset and participants with self-reported articular pain at 3 months post-infection. Both clinical markers may be useful in a health care setting as prognosis factors of chronic manifestations among adults with CHIKV infection.

The observed frequency of PA in our study was similar to estimates among individuals in Colombia at 6 months from acute disease onset (21) and adults on the island of Réunion at 1.5 years from acute disease onset (17, 18). The cumulative incidence at 15 to 36 months post-infection in our analysis was also lower than others previously published (15, 16, 20). Ethnic differences in pain perception and related conditions may be responsible for these differing findings (23), since pain sensitivity is characterized by individual and group variability (24, 25). These disparities have also been documented in musculoskeletal and chronic pain (26, 27).

Regarding age at disease onset, the cutoff used in our study (≥ 40 years of age) *Rev Panam Salud Publica* 41, 2017 corresponds to the median age of enrolled individuals. Similar findings were previously documented (15, 16, 20). We also observed a significant association between self-reported articular pain at 3 months from acute illness and PA at 6 months post-infection. A majority of individuals (58.8%) with self-reported arthralgia at 3 months persisted with pain at 6 months from the onset of acute disease. Another published study described a similar association between articular pain at 4 months post-infection and PA at 3 years from acute disease onset (16).

**TABLE 1:** Baseline characteristics of study sample in investigation of persistent arthralgia and related risks factors in laboratory-confirmed cases of Chikungunya virus infection, Mexico, 2015

Characteristic	*n* (%)
Sex	
Female	92 (67.7)
Age (years)^a^	42.4 ± 16.1
Age group (years)	
15 – 39	63 (46.3)
40 – 64	58 (42.7)
≥ 65	15 (11.0)
Days elapsed from disease onset to seeking medical attention^a^	1.8 ± 1.1
Ambulatory medical management	
Yes	136 (100.0)
Sites of articular pain at acute disease	
Hands	113 (83.1)
Wrists	118 (86.8)
Elbows	51 (37.5)
Shoulders	74 (54.4)
Neck	60 (44.1)
Back, upper	60 (44.1)
Back, lower	72 (52.9)
Hips	64 (47.1)
Knees	119 (87.5)
Ankles	115 (84.6)
Feet	110 (80.9)
Sites of articular effusion at disease onset	
None	28 (20.6)
Hands	72 (52.9)
Wrists	57 (41.9)
Elbows	15 (11.0)
Shoulders	10 (7.4)
Knees	57 (41.9)
Ankles	79 (58.1)
Self-reported recovery from symptoms of Chikungunya infection^b^	
No	75 (55.1)
Self-reported arthralgia during the past 8 days (any site)^b^^,^ ^c^	
Yes	57 (76.0)

***Source:*** This table was prepared by the authors from the results of research.

a The arithmetic mean ± standard deviation (SD) is presented.

b At 6 months from acute Chikungunya infection.

c Among participants who self-reported no recovery from symptoms of chikungunya infection.

We did not find an association between female sex and chronic articular pain. An increased risk of PA among females was previously observed (28). This positive association may be secondary to gender differences in the use of health services, since females traditionally tend to use them more frequently than do males, mainly due to reproductive-related causes and the care of children (29).

Neither type 2 diabetes mellitus nor high blood pressure had a significant association with increased risk of PA. Interestingly, the prevalence of these two chronic noncommunicable diseases in the study sample was higher than estimates from the 2012 National Health and Nutrition Survey (type 2 diabetes mellitus, 14.0% vs. 9.2%; arterial hypertension, 22.1% vs. 16.6%) in Mexican adults aged 20 years and older (30, 31).

More than one-third of participants with self-reported PA complained of severe arthralgia (NRS ≥ 7), which may be potentially incapacitating and which has been associated with deteriorated functional status (32). Therefore, the burden of disease of CHIKV-associated chronic manifestations may be considerable. The NRS has good sensitivity in pain evaluation (33).

We did not collect data regarding the pharmacological treatment of acute illness in our study. However, standardized diagnosis and treatment guidelines are followed in the health care setting where this research took place, and those procedures only include the prescription of acetaminophen and nonsteroidal anti-inflammatory agents (9).

In our study, a statistically significant sex-related difference was observed in the number of participants with self-reported severe pain at acute illness (81.8% for males vs. 95.7% for females, *P* = 0.008). Sex-related variability in pain sensitivity may explain this fact. The perception of pain is a complex phenomenon determined by biological factors and such psychosocial factors as gender role beliefs and pain-related expectancies (34). Among the individuals with PA, the prevalence of severe pain was 21.4% in males and 41.9% in females. This difference was not statically significant (*P* = 0.169), which could perhaps be due to a lack of power.

The underlying mechanism of CHIKV-associated PA has not been explained. Recent findings suggest that early infection of human monocytes by the virus is implicated in chronic arthralgia, since infected monocytes have been found in the synovial tissues of patients with PA (35, 36). Other histopathologic findings in synovial tissues include fibroblast hyperplasia, increased angiogenesis, high levels of matrix metalloproteinase-2, and acute cell death (36). The adaptive immune response seems to control the chronic CHIKV-associated rheumatic manifestations (37).

**TABLE 2:** Clinical markers among individuals with self-reported persistent arthralgia (PA) and those with full recovery at 6 months after Chikungunya virus infection, Mexico, 2015

Clinical markers	PA		Recovery		
	n	(%)	n	(%)	*P*^a^
Sex					
Female	43	(75.4)	49	(62.0)	0.099
Age (years)					
≥ 40	37	(64.9)	36	(45.6)	0.026
Severity of articular pain disease^b^					
Mild–moderate	5	(8.8)	7	(8.9)	0.986
Severe	52	(91.2)	72	(91.1)	
Length (days) of severe (incapacitating) articular pain					
1-14	22	(38.6)	43	(54.4)	0.130
15–30	12	(21.1)	9	(11.4)	
> 30	23	(40.4)	27	(34.2)	
Associated signs and symptoms					
Rash	50	(87.7)	62	(78.5)	0.163
Headache	49	(86.0)	40	(88.6)	0.646
Gastrointestinal manifestations^c^	27	(47.4)	28	(35.4)	0.162
Fatigue	73	(92.4)	55	(96.5)	0.318
Number of painful joints					
≥ 8	29	(50.9)	28	(35.4)	0.072
Articular effusion (any site)					
Yes	49	(86.0)	59	(74.7)	0.108
Personal history of					
Type 2 diabetes mellitus	9	(15.8)	10	(12.7)	0.603
Arterial hypertension	15	(26.3)	15	(19.0)	0.309
Osteoarthritis	9	(15.8)	8	(10.1)	0.324
Any chronic disease	28	(49.1)	28	(35.4)	0.110
Self-reported hyperuricemia					
Yes					
Articular pain at 3 months (any site)					
Yes	50	(87.7)	35	(44.3)	> 0.001
Sites of articular pain at 6 months from acute disease					
Hands	34	(59.6)	NA^e^	NA	
Wrists	27	(47.4)	NA	NA	
Shoulders	11	(19.3)	NA	NA	
Hips	8	(14.0)	NA	NA	
Knees	29	(50.9)	NA	NA	
Ankles	33	(57.9)	NA	NA	
Feet	34	(59.6)	NA	NA	
Other^d^	12	(21.1)	NA	NA	
Severity of articular pain at 6 months from acute disease					
Mild-moderate	36	(63.2)	NA	NA	
Severe	21	(36.8)	NA	NA	

***Source:*** This table was prepared by the authors from the results of research.

a *P* = *P* value from chi-square test.

b A self-report numeric rating scale (NRS) ranging from 0 to10 was used: mild – moderate, < 7; severe, ≥ 7.

c Gastrointestinal manifestations = diarrhea, vomiting, and abdominal pain.

d Other sites = elbows, neck, and back (upper/lower).

e NA = Not applicable

Biological markers that are typically altered in autoimmune rheumatic diseases are found within physiologic ranges among patients with long-term arthralgia after CHIKV infection (16).

Increased values of serum C-reactive protein (CRP) have been observed in subjects with PA (38).

Virus culture and isolation is the gold standard for diagnosis of CHIKV infection. The procedure, however, is cost-prohibitive due to the requirement of biosafety level 3 containment, and it may take one to two weeks to receive the results (1, 39). The RT-qPCR assay is a cost-effective alternative for the diagnosis of acute disease (40). Only cases of CHIKV infection confirmed by RT-qPCR were included in this analysis. During the disease outbreak, the RT-qPCR testing was performed at public health care facilities in 5% of the cases, which were randomly selected. The molecular assay is performed mainly for epidemiological purposes, according to normative guidelines (9). The Division of Laboratories for Surveillance and Epidemiological Research, where the assays were carried out, has high-quality standards and is endorsed by the federal Institute for Epidemiological Diagnosis and Reference (InDRE, *Instituto de Diagnóstico y Referencia Epidemiológicos)*. InDRE coordinates the laboratory-surveillance actions related to events of public health interest at the national level.

Although we had a binary outcome, RR was used as an association measure instead of odds ratio (OR) due to the longitudinal design of this study and the observed frequency of the event (41.9%). The odds may be biased when frequent events (> 10%) are evaluated (41, 42). Using a multiple logistic unconditional model, the OR of age ≥ 40 years old and of arthralgia at 3 months from acute disease was 2.26 (95% CI, 1.07-4.77) and 8.44 (95% CI, 3.20-22.28), respectively. These ORs are higher than the corresponding RRs (for age, RR = 1.60; 95% CI, 1.03-2.48; for arthralgia at 3 months from disease onset, RR = 3.95; 95% CI, 1.95-8.01).

In our study, only individuals affiliated with the IMSS were enrolled, and the study sample was not representative of the general population. However, nearly 45% of the total population of the state of Colima is insured by this health institution, and the demographic and socioeconomic profile of its users is heterogeneous. We do not consider that the lack of representativeness had any influence on our findings. Additionally, methodological data regarding observational epidemiologic studies suggest that scientific inference does not stand in need of representativeness of the general population in order to be valid (43, 44). Therefore, the overall effect of a specific exposure may result from an average effect that has been weighted by the distribution of individuals across subgroups (43).

**TABLE 3:** Bivariate and multiple analysis, with risk ratios (RRs) and 95% confidence interval (CIs), between clinical markers and persistent arthralgia at 6 months from laboratory-positive Chikungunya infection, Mexico, 2015

Clinical markers	Bivariate analysis			Multiple analysis		
	RR	95(%)CI	P	*RR*^a^	95(%)CI	P
Sex						
Male	1.00	1.00	
Female	1.47	0.90–2.39	0.121	1.20	0.77–1.89	0.416
Age (years)						
< 40	1.00	1.00	
> 40	1.60	1.04–2.45	0.032	1.60	1.03–2.48	0.037
Sites (no.) of articular pain at acute disease						
> 8	1.00	1.00	
< 8	1.44	0.97–2.13	0.072	1.24	0.88–1.74	0.225
Articular effusion (any site) at acute disease						
No	1.00	1.00	
Yes	1.59	0.85–2.96	0.146	1.44	0.76–2.71	0.260
Length (days) of severe articular pain at acute disease	1–14	1.00	1.00	
15–30	1.69	1.02–2.79	0.042	1.27	0.79–2.05	0.319
> 30	1.36	0.86–2.14	0.187	0.89	0.60–1.32	0.557
Arthralgia (any site) at 3 months from acute disease						
No	1.00	1.00	
Yes	4.29	2.10–8.75	< 0.001	3.95	1.95–8.01	< 0.001
History of ≤ 1 chronic disease^b^						
No	1.00	1.00	
Yes	1.38	0.93–2.04	0.108	1.13	0.68–1.88	0.648

***Source:*** This table was prepared by the authors from the results of research.

a The relative risks from multiple analysis are adjusted by the variables listed in the table.

b Chronic disease = type 2 diabetes mellitus, arterial hypertension, or osteoarthritis.

### Conclusions

Long-term manifestations associated with CHIKV infection seem to be a frequent event in the study population. To the best of our knowledge, this is the first report on this phenomenon in this specific population.

Two independent risks factors were associated with increased risk of PA after 6 months from acute disease onset: age (≥ 40 years) and self-reported arthralgia at 3 months post-infection. Among individuals with laboratory-confirmed infection, the risk stratification of PA may be useful for identifying patients in whom an early medical intervention would be beneficial in reducing the associated disease burden.

#### Funding.

*(Consejo Nacional de Ciencia y Tecnología)* (CONACyT) Ph.D. scholarship received by EMZ.

#### Disclaimer.

Authors hold sole responsibility for the views expressed in the manuscript, which may not necessarily reflect the opinion or policy of the *RPSP/PAJPH* or PAHO.
